# Vascular Tortuosities of the Upper Eyelid: A New Clinical Finding in Fabry Patient Screening

**DOI:** 10.1155/2013/207573

**Published:** 2013-10-07

**Authors:** Langis Michaud

**Affiliations:** École d'Optométrie de l'Université de Montréal, 3744 Jean Brillant, Suite 190-70, Montreal, QC, Canada H3T 1P1

## Abstract

*Purpose*. To report a new clinical finding related to Fabry disease. *Methods*. Fabry subjects were enrolled in the study, matched for age and sex with healthy individuals as a control group. This is a prospective review of all upper lid pictures taken for every subject at their last visit. A 4-step grading scale is proposed to classify this new entity. *Results*. Group A (Fabry) comprised 16 males and 22 females, aged 40 (±14) years on average. Group B (control) comprised 7 males and 8 females, aged 37 (±12) Vessels tortuosity was identified on the external superior lid in 36 of the Fabry patients (94.7%), while none of the subjects in group B showed similar vessels tortuosity. In addition, microaneurysms (MAs) were found in 10/38 group A subjects while none in group B presented a similar finding. The differences are highly significant. *Conclusion*. This paper proposes that blood vessels tortuosity on the upper eyelid be recognized as a new clinical entity for inclusion among the classic ocular manifestations of Fabry's disease. Without evidence of any negative impact, it should be considered a benign sign contributing to evidence of suspected Fabry disease.

## 1. Introduction

With an incidence varying from 1/3,000 newborn males to 1 : 40,000 or 1 : 117,000 live male births [1], Fabry is considered one of 7,000 known rare diseases that exist in the USA and elsewhere [2]. However, the number of patients is probably underestimated [3], because their symptoms are quite variable and unspecific, often leading to confusion with rheumatoid diseases or chronic inflammatory conditions [4]. A confirmatory diagnosis comes, on average, 14 years after the onset of the first symptoms for male adults and 16 years for females [5], three years for symptomatic children [6]. Considering the life-threatening aspect of the disease, this delay is unfortunate. Because ocular manifestations are among the first to appear in Fabry subjects [7], eye care practitioners are key players in detecting and screening for this disease.

This paper aims to present a new clinical finding that may help them improve screening effort and alert them to potential Fabry subjects while performing slit lamp examinations. It also underscores the importance of examining the external surface of the upper lid, an area usually hidden and left unexplored during routine anterior segment examination of the eye. 

## 2. Subjects and Methods

The Université de Montréal, École d'Optométrie, is conducting a longitudinal study on ocular manifestations related to Fabry disease. Details of the study design and methods have been published elsewhere [8] but are briefly described here. Since 2010, thirty-eight Fabry subjects have enrolled in the study, matched for age and sex with 15 healthy subjects acting as a control group. Enrollment required a confirmed diagnosis of Fabry disease and consent to be seen on an annual basis for five years. For the control group, subjects had to be healthy, nonmedicated, and free of any ocular pathology at the moment of enrollment. The study was approved by the Institutional Ethics Committee and respects the tenants of the Declaration of Helsinki.

Annual examination of both groups includes several refractive and ocular health tests, including a slit lamp examination, gonioscopy, high order aberration evaluation, corneal hysteresis measurement, fundus and anterior segment photography, OCT assessment of the optic nerve, and threshold visual fields. At the very beginning of the longitudinal study, one subject showing a high sensitivity to light closed his eyes during a routine slit lamp procedure. The author then observed abnormal blood vessel tortuosity and microaneurysms on the external part of the upper lid. Based on this anecdotal finding, all other subjects since then have been screened to confirm the presence of vessel tortuosity and/or microaneurysms affecting this area.

This report analyzes the results of anterior segment photography from the last examination of every subject. The slit lamp used for this study was a Topcon SLD-7. Photos were taken with a Nikon D-100 camera mounted on the slit lamp. Images were kept and saved on a computer using Image 2000 software provided by Topcon Canada (Montreal, Quebec). In cases where multiple pictures were taken for the same patient, the image with the best definition was kept. All of the images were taken in diffuse lighting, with an illuminated background. They were magnified for the next analysis. Subjects were asked to close their eyes. The upper lid area, from nasal to temporal sides, was carefully examined. Any vessel tortuosity and/or microaneurysms were noted and documented by the same photographer for every patient. For some of them, vessels were best seen when the upper lid was stretched.

The images were analysed by the author. They were classified into 4 groups based on the severity of the tortuosity observed: none, mild, moderate, and severe (defined in Table 1). Because this entity has never been reported before, a grading scale was developed using the Canadian Fabry Disease Initiative (CFDI) [9] grading scale for conjunctival vessel tortuosity for reference purposes, given that it reflects an international consensus. 

## 3. Results

Group A comprised 38 Fabry subjects: 16 males and 22 females, aged 40 (±14) years old on average. A majority of male subjects (12) and a fewer females (7) were undergoing enzyme replacement treatment at the time of the visit. Group B comprised 15 healthy subjects: 7 males and 8 females, aged 37 (±12) years old. Male subjects in general and those under enzyme replacement therapy in particular were found to be more symptomatic than female ones. Male patients were more likely affected by angiokeratomas (treated 91.7%/nontreated 71.5%), acroparesthesia (83.3% versus 50%) and tinnitus (66.6% versus 50%), or renal dysfunctions (58.3% versus 50%). Female patients were found with cardiopathy (treated 85.7% versus nontreated 33.3%), angiokeratomas (71.4% versus 13.3%), and acroparesthesia (57% versus 53.3%). Gastrointestinal problems and fatigue were more common in females without enzyme replacement treatment. It is important to note that overall, one out of two patients was affected by fatigue/depression. About a third of them are also complaining about vertigo and respiratory problems (clinical signs and symptoms of group A cohort are summarized in Table 4).

**Figure 1:**
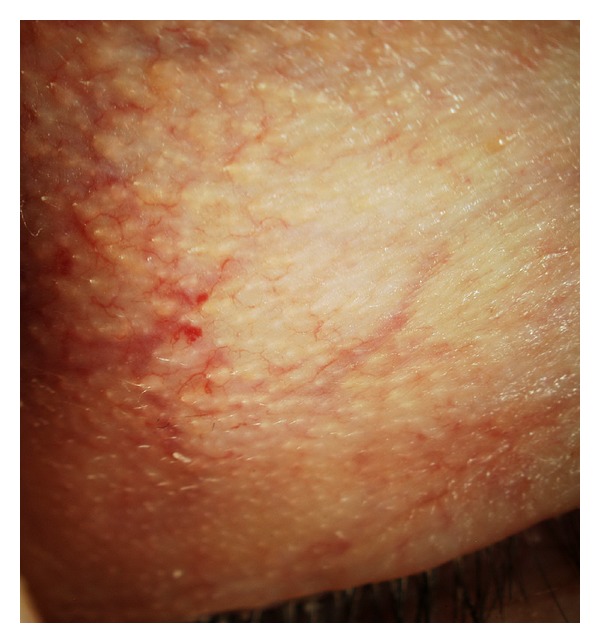
Vascular Tortuosity of the Upper Eyelid (VTUE) severe grade with telangiectasia and microaneurysms.

**Figure 2:**
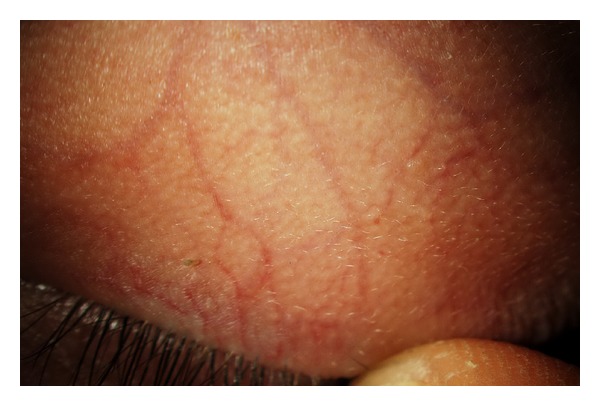
Low grade of blood vessels tortuosity as seen on the upper lid of a Fabry patient.

**Figure 3:**
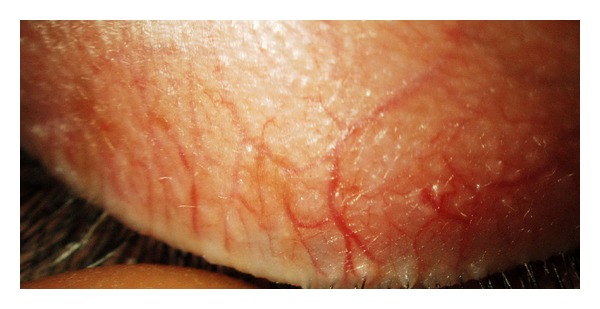
Moderate grade of blood vessels tortuosity.

**Figure 4:**
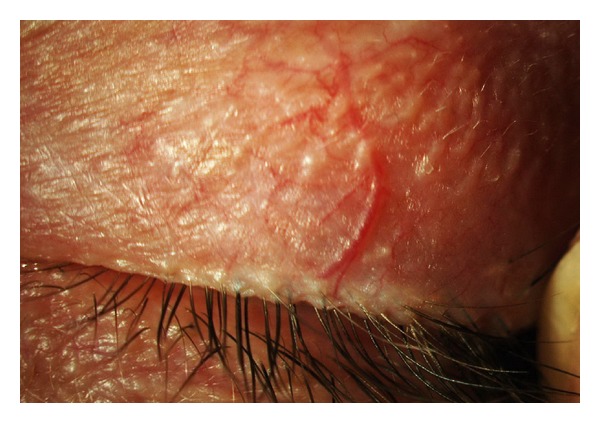
Moderate grade with a higher vessels tortuosity.

**Figure 5:**
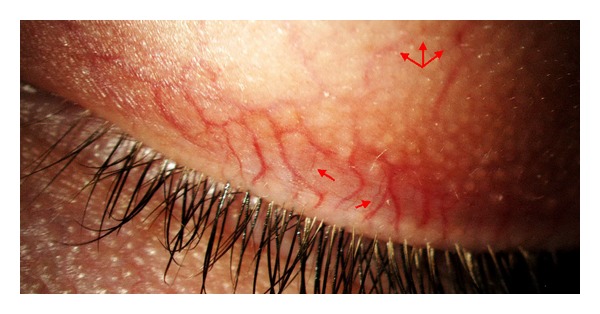
Severe grade of blood vessels tortuosity with presence of microaneurysms and telangiectasia (arrows).

**Figure 6:**
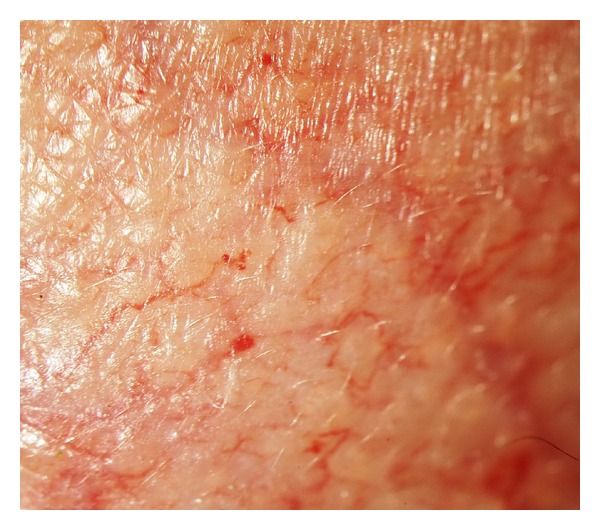
Higher magnification of microaneurysms seen with the severe form of vessels tortuosity.

Ocular photography of the upper lid of both eyes was obtained from both groups. Blood vessels tortuosity was identified in 36/38 of the Fabry patients (94.7%), while none of the subjects in group B showed similar finding (see Table 2 and Figure 1). Male subjects and those under treatment were more likely to show severe tortuosities. In some cases, group B subjects had blood vessels visible under the slit lamp. However, the vessels observed in group B subjects were straight, nontortuous, nondilated, and free of telangiectasia and were located deeper in the skin (less visible). In addition, microaneurysms (MAs) were found in 10/38 group A subjects (see Table 3 and Figures 2, 3, 4, 5 and 6) while none in group B presented a similar finding. Globally, 82.5% of the subjects presenting MA on the upper lid showed the same finding for bulbar conjunctiva. Statistically, the differences observed between the 2 groups were highly significant (Wilcoxon test, *P* < 0.005). 

## 4. Discussion

To our knowledge, this is the first time that the presence of blood vessel tortuosity visible on the external side of the upper lid has been reported in Fabry patients. It was probably not identified before because this area is rarely assessed during routine eye examinations. Individuals under treatment are more likely to show severe manifestations of vessels tortuosity, in the lids and elsewhere in the body. Males are also more likely to be affected than females. Such modification of the vascular tissue is not surprising considering the nature of Fabry disease.

### 4.1. Nature of the Disease

Fabry disease is an X-chromosome linked disease including 431 different mutations for the GLA gene [10]. It is characterized by a deficiency of the lysosomal enzyme alpha-galactosidase A (GLA or *α*-gal A) [11], secondary to protein misfolding and/or an inability to direct the enzyme towards the lysosomes. In Fabry, the enzyme deficiency leads to an accumulation of substrate within the cells, with a preference for organs that naturally accumulate the greatest amount (the heart and kidneys) [12]; it also favours the vascular endothelial cells, renal dorsal root ganglion cells, the cornea, and the skin [13]. This is why the observation of vascular changes on the palpebral area is quite likely. 

### 4.2. Ocular Manifestations

Ocular manifestations resulting from glycosphingolipid deposits are among the first to occur in life. They can be identified very early in childhood, by age three [14] if not earlier [15]. They usually occur at the same time as systemic symptoms, especially in hemizygous subjects. 

Classic ocular manifestations include vortex pigmentation of the cornea (verticillata), lens opacities (anterior whitish opacities and posterior subcapsular cataract), conjunctival vessel anomalies, and retinal vessel tortuosities [14], although such manifestations do not usually impair vision or create visual symptoms. 

Our findings suggest that upper lid blood vessels tortuosity must be added to the list of key ocular manifestations of Fabry disease, as it is the case for other tortuosities affecting the eye. From the data presented, it is quite clear that most of group A subjects (94.7%) were affected by blood vessel tortuosity similar to that found in the conjunctiva and elsewhere in the eye and their body (angiokeratomas, see Table 4). Males, under treatment, showed the most severe forms of vessels tortuosity, followed by females under treatment and then males and lastly females, both untreated. This confirms previous results with respect to conjunctival vessel abnormalities [14]. 

The only unique feature found on the lid, compared to the conjunctiva, was the presence of telangiectasia. This sign is habitually present when new blood vessels are generated in response to a lack of oxygen perfusion. The presence of the substrate within the blood vessels can contribute to alter the perfusion of the oxygen to the surrounding tissue. Angiogenic factors can consequently be released, leading to the development of new blood vessels.

Based on their clinical features, vessels tortuosity observed in Fabry subjects cannot be confused with any kind of inflammation of the lid (blepharitis). In addition to the absence of characteristic clinical findings related to lid inflammation, subjects did not complain of acute redness, lid swelling, debris, deposits, foreign body sensation, or lids stuck in the morning. Clinical findings were found involving most of the area of the upper eye lid surface, not only the lid margins, contrary to other lid pathologies such as meïbomitis or posterior blepharitis (ocular rosacea). There was no report of lid surgery in any case. 

The findings were quite different for group B subjects. Blood vessels on normal subjects are visible but do not present tortuosities, microaneurysms, nor telangiectasia. They are less visible and located deeper in the tissue. 

Because this study is one of the few that have gathered together such a large number of Fabry patients and because upper lid blood vessels tortuosity was observed in over 90% of these subjects, we are extremely confident in proposing this clinical feature as a characteristic of the disease. 

Based on the case of other tortuosities observed in Fabry, we suspect that their likely cause is the accumulation of globotriaosylceramide (GL3) within the endothelial blood vessel cells. As it is for the retina, we do not expect any long-term negative impact from the presence of vessel abnormalities on the upper lid. There is no evidence of any resulting mechanical or functional dysfunction. Only long-term observation will tell if such tortuosities have the potential to affect ocular health in any manner. 

### 4.3. Limitations of This Study

The first element to consider as a bias in this study is the number of subjects recruited as control (group B), being half of the Fabry subjects (group A). Limited manpower to recruit and follow subjects explains in part the reason for this discrepancy. However, we do not feel that this factor can influence the outcome of the study considering the high number of subjects showing blood vessels tortuosities in group A while none showed the same finding in group B. Even with more subjects in group B, the possibility to find a difference in the number of suspects with the same vessels tortuosity is minimal. 

A second bias can come from the fact that the study of the images was made by the same investigator who did the examination. It is true that analysis by a second reader, blinded to the subjects, would add some value to the findings. However, results are so obvious that the necessity to rely on an external validation appears to be limited in regard to the clinical findings found. 

Lastly, the observational nature of this study can be discussed. We were obviously unable to perform histological comparisons of the two groups that participated in this study. It would be interesting, at some point, to investigate the cellular level in order to establish anatomical evidence of the blood vessel tortuosity characteristic of Fabry's disease in the affected subjects. 

## 5. Conclusion

This paper proposes that blood vessels tortuosity, visible on the external surface of the upper eyelid, be recognized as a new clinical entity for inclusion among the classic ocular manifestations of Fabry's disease. To classify this new entity, a four-step grading system is proposed. Without evidence of any negative impact, it should be considered a benign sign contributing to evidence of suspected Fabry's disease, especially in male patients. 

## Figures and Tables

**Table 1:** Grading scale proposed for blood vessels tortuosity of the upper eyelid.

Grade	Vessels tortuosity	Microaneurysms
1 (none)	No tortuosity nor vessels abnormality seen	None

2 (low)	Dilated, prominent vessels, at the surface, non-tortuous, in limited areas (<50%) of the superior lid	None

3 (moderate)	Dilated, tortuous vessels, affecting >50% of the superior lid surface. Telangiectasia can be seen.	Present (at least 1)

4 (severe)	Dilated, tortuous vessels, extending from nasal to temporal side. Telangiectasia is present.	Present (2 or more)

**Table 2:** Presence and severity of blood vessels tortuosity among group A (Fabry) subjects.

	OD				OS			
	Male Tx (*n* = 12)	Male no Tx (*n* = 4)	Female Tx (*n* = 7)	Female no Tx (*n* = 15)	Male Tx (*n* = 12)	Male no Tx (*n* = 4)	Female Tx (*n* = 7)	Female no Tx (*n* = 15)
Grade 1 (none)	0	0	0	2	0	1	0	2
Grade 2 (low)	1	2	2	13	3	0	4	12
Grade 3 (moderate)	8	1	3	0	7	2	2	1
Grade 4 (severe)	3	1	2	0	2	1	1	0

**Table 3:** Presence and location of microaneurysms in group A (Fabry) patients.

Micro-aneurysms	OD (*n* = 38)		OS (*n* = 38)	
	Nasal	Temporal	Nasal	Temporal
Conjunctival	7 (18.6%)	6 (33.3)	4 (10.5%)	8 (21.1%)
Palpebral	5 (13.1%)	5 (13.1%)	4 (10.5%)	6 (33.3%)
% palpebral versus conjunctival	5/7 = 71.4%	83.3%	100%	75.0%

**Table 4:** Reported symptoms of Fabry patients.

	Male (% of patients)		Female (% of patients)	
	Under treatment (Tx)	Nontreated (no TX)	TX	No TX
Acroparesthesia	**83.3**	**50**	**57.1**	**53.3**
Cardiopathy (blood hypertension, abnormal ECG)	58.3	25.0	**85.7**	33.3
Renal dysfunction: abnormal creatinine	58.3	**50.0**	42.9	26.7
Hypohidrosis	58.3	0.0	14.2	33.3
Intolerance to cold and heat	25.0	0.0	14.2	33.3
Gastrointestinal problems	33.3	0.0	42.9	**46.7**
Angiokeratomas	**91.7**	**75.0**	**71.4**	13.3
Tinnitus	**66.6**	**50.0**	14.2	20.0
Fatigue	41.7	**50.0**	42.9	**46.7**
